# Adherence to hypertension medication: Quantitative and qualitative investigations in a rural Northern Vietnamese community

**DOI:** 10.1371/journal.pone.0171203

**Published:** 2017-02-01

**Authors:** Thi-Phuong-Lan Nguyen, Catharina C. M. Schuiling-Veninga, Thi Bach Yen Nguyen, Thu-Hang Vu, E. Pamela Wright, Maarten J. Postma

**Affiliations:** 1 Department of Pharmacy, Unit of PharmacoTherapy, -Epidemiology & -Economics (PTE2), University of Groningen, Groningen, the Netherlands; 2 Department of Health Economics, Ha Noi University of Medicine, Ha Noi, Vietnam; 3 Thai Nguyen University of Medicine and Pharmacy, Thai Nguyen, Vietnam; 4 Medical Committee Netherlands-Vietnam, Amsterdam, The Netherlands; 5 Institute of Science in Healthy Aging & health caRE (SHARE), University Medical Center Groningen (UMCG), University of Groningen, Groningen, the Netherlands; 6 Department of Epidemiology, UMCG, University of Groningen, Groningen, the Netherlands; University of Miami School of Medicine, UNITED STATES

## Abstract

**Objectives:**

The purposes of this study were to assess the adherence to medication of hypertensive patients visiting community health stations in a rural area in Vietnam, to examine the relationship between levels of adherence and cardiovascular risk among hypertensive patients and to further understand factors influencing adherence.

**Methods:**

This study is part of a prospective one-year study conducted on hypertension management in a population aged 35 to 64 years. Data on age, sex, blood pressure and blood test results were collected at baseline. Cardiovascular risk was based on the Cardiovascular Risk Prediction Model for populations in Asia. To calculate medication adherence, the number of days the drug was taken was divided by the number of days since the first day of the prescription. A threshold of 80% was applied to differentiate between adherence and non-adherence. In-depth interviews were conducted among 18 subjects, including subjects classified as adherent and as non-adherent.

**Results:**

Among 315 patients analyzed, 49.8% of the patients were adherent. Qualitative investigation revealed discrepancies in classification of adherence and non-adherence based on quantitative analysis and interviews. No significant difference in medication compliance between two cardiovascular disease risk groups (<10% vs. >10% risk) was found, also not after controlling for age, sex, and ethnicity (adjusted odds ratio at 1.068; 95% CI: 0.614 to 1.857). The odds of medication adherence in females was 1.531 times higher than in males but the difference was not statistically significant (95% CI: 0.957 to 2.448). Each one-year increase in age resulted in patients being 1.036 times more likely to be compliant (95% CI: 1.002 to 1.072). Awareness of complications related to hypertension was given as the main reason for adherence to therapy.

**Conclusions:**

Medication adherence rate was relatively low among hypertensive subjects. The data suggest that rather than risk profile, the factor of age should be considered for guiding the choice on who to target for improving medication adherence.

## Introduction

Many studies have suggested that a high level of adherence to antihypertensive drug treatment is related to better blood pressure (BP) control and a reduced risk of cardiovascular disease (CVD) [1–15]. The scarce studies available so far suggest that adherence to antihypertensive medication is often relatively low. A meta-analysis on data of 376,162 American patients showed an adherence to medication for preventing cardiovascular disease of only 57% [16]. Similarly, a study conducted in Italy showed that approximately 60% of the patients had a good-to-excellent adherence to antihypertensive medication [2], whereas in Poland only 26% of the cardiovascular patients used their drugs as prescribed [17]. Many Asian studies mimic this same trend of low medication adherence; the percentage of patients showing good adherence was 53% in Malaysia [8], 65% among Chinese populations [18], 55% in Korea [19] and 66% in Vietnam [20].

Knowledge of factors that affect adherence could play an important role in the development of interventions to improve it. From both qualitative and quantitative studies described in the literature, many factors potentially affecting adherence to medication are known, including demographic, social and cognitive factors, interactions between health care providers and patients, health care system characteristics, the medication involved, and the general health profile of the patient [14, 16, 20–33]. For instance, one meta-analysis showed that patients with a history of CVD were more often adherent to medications such as aspirin, BP-lowering drugs and statins, compared to patients without previous CVD [16]. Another Polish study conducted at primary care level revealed that medication adherence was higher in patients at lower level of CVD risk, and that there was a weak correlation between CVD risk level and non-adherence to medication [17].

As there is a lack of evidence on the association between CVD risk and the adherence to antihypertensive medication in general and in developing countries such as Vietnam in particular, we conducted this study to (i) assess the level of adherence of hypertensive patients visiting community health stations (CHS) in a rural area in Vietnam; (ii) examine the relationship between level of adherence and cardiovascular risk among hypertensive patients; and (iii) get a better understanding of adherence and factors influencing adherence among these patients. Information on adherence is crucial for estimating the effectiveness of antihypertensive drugs, in addition to data on efficacy from the clinical trials. Together with data on the costs of screening and treatment, these findings will provide input parameters for future modeling in a full-fledged cost-effectiveness study of community programs for the control of hypertension in Vietnam.

## Methods

### Study design and setting

This study was conducted in rural mountainous communes in the North of Vietnam among subjects aged from 35 to 64 years. Four districts were selected purposively. We randomly selected people in the desired age range from enough villages to cover 45% to 50% of the population in each commune, and one commune per district among four districts in Thai Nguyen province. The selection of study locations and the baseline surveys have been described in detail elsewhere [34]. Both quantitative and qualitative methods were applied in order to get evidence on both the level of medication adherence and factors potentially explaining adherence or non-adherence. Notably, the quantitative study used a prospective design with a 1-year time frame and the qualitative study involved an in-depth interview 18 months after the start of the baseline survey. The quantitative study included the data on medication use and adherence and patient characteristics potentially associated with adherence. The in-depth interviews allowed us to get further understanding of factors influencing the level of adherence. Factors that have been suggested in the literature to influence adherence are listed in Table 1 [14, 16, 20–33, 35]

**Table 1:** Factors potentially affecting medication adherence reported in the literature.

Group	Factors
Demographic factors	
	Age
	Sex
	Race
	Education level
Social factors	
	Social economic status
	Social support
Cognitive factors	
	Understanding of cause and effect of hypertension
	Awareness of hypertension risk
	Awareness of BP target and medication indication, forgetfulness and self-efficacy and sensing timing to take medication
Health care system characteristics	
	Communication between providers and patients
	Frequency of visits to health-care providers
	Availability of spare time to see doctor
	Quality of communication when in the office
Health care system characteristics	
	Health insurance
	Health care system type
	Providers’ typology
Medication involved	
	Inclusive drug class
	Multiple/single dosage
	Complexity of regimen
	Potential and actual side-effects
	Shortages of drugs
	Total number of pills per day
General health profile of the patient	
	History of cardiovascular disease
	Comorbidity
	Depression
	Exact BP level
	Possible symptoms of hypertension
	Quality of life

### Quantitative study

#### Patient selection

Patient selection at the time of the baseline survey has been described in detail elsewhere [34]. In brief: based on the list of population in each location, we invited people from 35 to 64 years old to participate in the study if they were not under current treatment for chronic diseases such as diabetes, hypertension, cancer, HIV/AIDS or heart disease. In each location, we measured the blood pressure of all participants to identify subjects with hypertension according to the Seventh Joint National Committee on Hypertension Clarification [34, 36]. After the baseline survey, subjects were either advised to manage their BP at a CHS or referred to second-line healthcare services. The latter choice was made if the local physician could not clearly identify a cardiovascular issue or suspected a potentially serious health condition better treated in hospital. We followed up those subjects who met the following inclusion and exclusion criteria. The main inclusion criteria were that subjects must have had a medication prescription for at least one month and had at least 90 days of follow up since the first prescription. Exclusion criteria were: (i) history of myocardial infarction or other serious heart disease(s), or any heart diseases which need to be treated in second-line facilities; (ii) referral to second-line if, despite strictly following the prescribed regimen, BP was inadequately controlled or organ damage was suspected; (iii) referral to second-line because patients requested it, generally thinking that their hypertension would be better managed there; (iv) patients had moved to another place to live; (v) patients no longer needed to take anti-hypertensive drugs; and (vi) patients missed getting a prescription for two months or more between two doses, because their BP had decreased to below 140 mm Hg during that time and their physicians had decided they could stop medication. We recruited participants with these inclusion and exclusion criteria because we believed them to be actual hypertensive patients who could continue to manage their blood pressure at the CHS and could be followed up.

#### Follow-up

Medical doctors at participating CHSs attended a 3-day training program in hypertension management conducted by a cardiologist from Thai Nguyen University of Medicine and Pharmacy. Subjects managed at the CHS were advised to visit the CHS every month to check their BP and receive anti-hypertensive drugs if needed. Subjects were also advised to visit the CHS more frequently if monitoring of BP or symptoms was needed, especially in cases where the types of medicine were changed. Subjects were advised to quit smoking, reduce salt intake, stop drinking alcohol, and of course, to take the drugs as prescribed. We followed subjects for one year after the date of registering at the CHS and recorded their BP and drug prescriptions at every visit. Analysis of adherence was based on collecting prescriptions.

#### Variables, measurements and statistical analyses

In a previous paper, we suggested that the Asian or Chinese risk models could be used to predict CVD risk in South-East Asia [34]. In this study, we applied the Asian model because it was developed using data from six cohorts in Asia [37] and therefore likely more representative for non-Chinese Asian countries, such as Vietnam. Input data for this model were collected at baseline, including systolic BP, cholesterol level, smoking status, age and sex of subjects. Subsequently, we divided the subjects into two groups: those having less than 10% and those having more than 10% risk of CVD in the coming eight years according to the Asian risk model. Detailed measurements of all variables were described in a previous report [34].

To assess the level of adherence to antihypertensive medication, we applied the interval-based method [38]. The number of pill-days covered was calculated; i.e., the number of days the drug was taken divided by the total number of days since the first day of prescribing. A threshold of 80% was applied to differentiate between adherence and non-adherence [39–41].

We used Chi square and Wilcoxon tests to examine whether adherent and non-adherent patients differed with regard to their CVD risk, sex and age. We then used logistic regression to investigate whether adherence could be predicted by CVD risk, sex, age and ethnicity. Continuous variables were presented as means ± standard deviation (SD) and non-continuous variables were presented as percentages.

### Qualitative study

For the qualitative study, we used purposive sampling to select both adherent and non-adherent subjects from two different communes. Information on adherence came from the quantitative study. The number of subjects interviewed was determined by the results of the interviews and decided by the two main researchers, based on all notes after each interview. New subjects were interviewed until the point of saturation, when no extra information was collected with additional interviews.

Three interviewers were trained by the primary researcher. The face-to-face interviews lasted 30 minutes and were conducted using a semi-structured questionnaire, in a private room at the CHS. Each interview was recorded and later transcribed. The main topics of the interview included: (i) drugs used; (ii) reasons for (non-)adherence; (iii) side effects; and (iv) information received from physicians at baseline. The in-depth interview guideline is presented in full in S1 Appendix.

For data analysis, two researchers read all 18 transcripts to understand the whole situation and subsequently we did data extraction to identify and group meaningful statements according to the main topics. Adherence in the qualitative part was also assessed using the cut-off point at 80%, but here we tried to relate this directly to the self-reported use of medications by the patients. For example, from the qualitative study adherence was considered as missing no more than one day per week on a regular basis, according to self-reporting during the interview. Non-adherent subjects were those who reported that they had stopped taking their medication, or took the medicines less than 20 days per month, or forgot their medicines on average two or more days per week.

### Human subjects and ethical issue clearance

The research proposal was approved by the Institutional Review Board in biomedical research in the Institute of Social and Medical Studies in Hanoi, Vietnam. Written informed consent was collected at the time of the baseline survey. Study subjects received a small compensation for their time at every visit (25,000 VND, about 1.20 USD).

## Results

### Quantitative results

**Fig 1:**
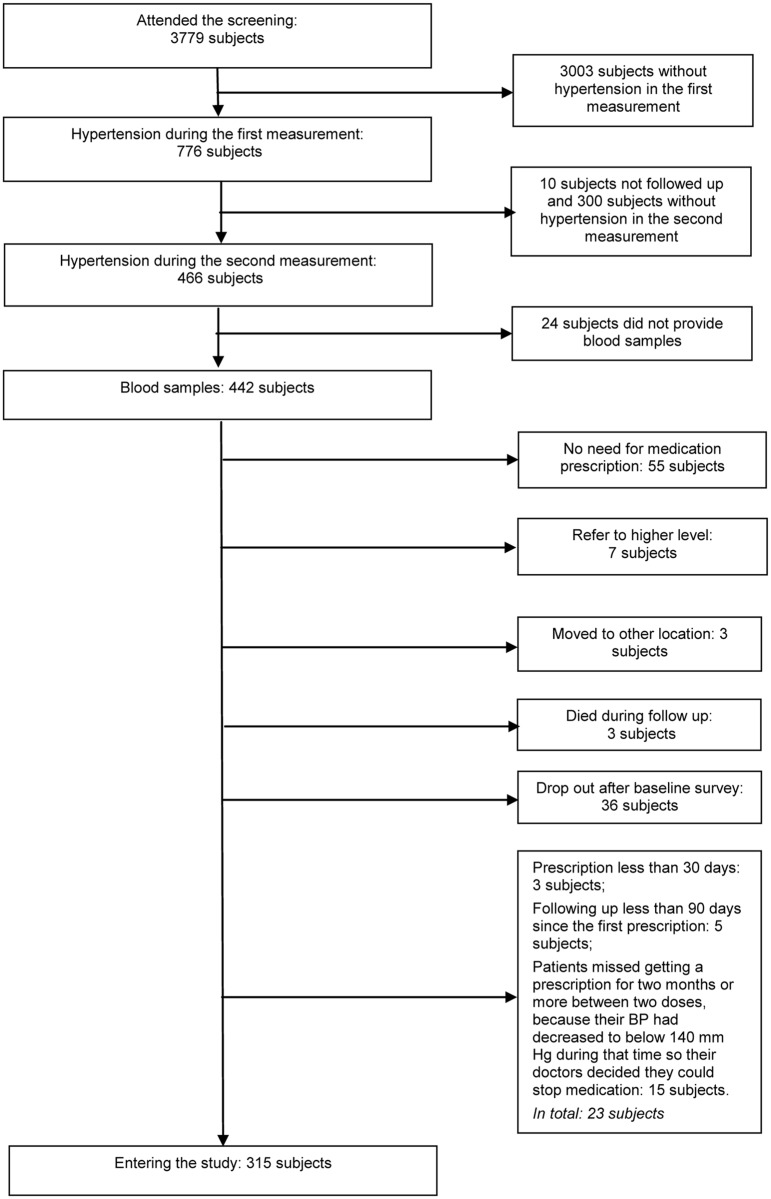
Flow chart of selecting patients after baseline survey.

As shown in Fig 1, 315 subjects entered into the one-year prospective study. Table 2 presents the characteristics of the subjects: 54% males and 82% ethnically Kinh (the majority ethnic group in Vietnam). The mean age was 53.7 years and the CVD risk—measured according to the Asian risk model—was less than 10% in the coming eight years for 76% of the subjects.

**Table 2:** Characteristics of patients (n = 315).

Characteristic of patients	n (%)	Adherence n (%)	Non-adherence n (%)
**Sex**			
Male	171 (54.3)	77 (45.0)	94 (55.0)
Female	144 (45.7)	80 (55.6)	64 (44.4)
**Ethnic**			
Kinh	259 (82.2)	136 (52.5)	123 (47.5)
Others	56 (17.8)	21 (37.5)	35 (62.5)
**% CVD risk**			
<10%	240 (76.2)	119 (49.6)	121 (50.4)
≥10%	75 (23.8)	38 (50.7)	37 (49.3)
**Mean age (years +/- SD)**	53.7 +/- 6.95	54.6	52.8

In the CHS setting, 49.8% of the patients were adherent to their antihypertensive medication.

As presented in Table 3, age did differ significantly between adherent and non-adherent subjects. Each one year increase in age resulted in subjects being 1.04 times more likely to be adherent (95% CI from: 1.002 to 1.072; p = 0.04). We found no association between CVD risk (<10% vs. ≥ 10% risk) and being adherent (odds ratio at 1.07; 95% CI from 0.61 to 1.86; p = 0.81). The difference between males and females in being adherent was not significant (95% CI: 0.96–2.45; p = 0.07).

**Table 3:** Associations (COR, AOR*, CI, p) of (non)adherence and patients’ characteristics.

Parameters	Adherence vs. non-adherence			
	Crude analysis		Adjusted analysis	
	COR (95% CI)	p	AOR (95% CI)	p
**CVD risk**				
CVD risk <10% (ref)	1		1	
CVD risk ≥ 10%	1.044 (0.622–1.754)	0.89	1.068 (0.614–1.857)	0.815
**Sex**				
Male (ref.)	1		1	
Female	1.526(0.977–2.383)	0.071	1.531 (0.957–2.448)	0.076
**Ethnic**				
Kinh (ref)	1		1	
Other	0.584 (0.32–1.067)	0.09	0.599 (0.328–1.095)	0.096
**Age (years)**		0.02	1.036 (1.002–1.072)	0.036

Note: Binary logistic regression, adjusting for the other factors shown in the table (CVD risk, sex, ethnic, age). COR: crude odd ratio; AOR: Adjusted odd ratio; CI: confidence interval.

### Qualitative results

To deepen our understanding of the background of adherence versus non-adherence, we interviewed 18 subjects (12 males), of whom 11 had been identified as adherent based on the criteria and data in the quantitative study.

Interestingly, a range of reasons for adherence and non-adherence were mentioned. Being aware of the complications of high BP or experience within the family with complications was mentioned as a factor enhancing good adherence. Most of the occasions when patients did not take the medicine were related to forgetting, when they were busy or changed their daily activity pattern; only two of 12 actively decided not to take the pills because they felt better. For example, one man said: “*I reduced the number of pills*…*and I did not see different results*”. Side effects appeared as another influential factor; six subjects had side-effects during the treatment, such as cough, headache, nausea or fatigue, which might have led to interruptions in taking medicines. Subjects explicitly recounted: *“After taking the first pills*, *I felt serious headaches…*. *so I stopped taking the medicines*,….*no one advised me*,….*I waited until next month to see doctor then she changed medicine for me”* or *“I got headache*….*then I changed the medicine”*.

Notably, the adherence classification from the quantitative results, based on follow-up data, was sometimes inconsistent with the information reported during interviews. Two of the 11 quantitatively adherent subjects reported periods of non-adherence, while four of the seven quantitatively non-adherent subjects could be classified as adherent based on the interviews. Five of the 11 adherent subjects stated in the interview that they had never forgotten to take the medicines, only maybe sometimes changed from morning intake to afternoon intake. Of the 12 subjects who mentioned having forgotten to take the medicines, two took the medicine still within a day but at a different time, while the others did not take the drug at all that day. Two subjects classified as non-adherent by the follow-up quantitative study, reported in interviews that during some months that they didn’t get medicines from the CHS, they bought the medicines themselves (this is possible in the open market for pharmaceuticals in Vietnam).

## Discussion

The results of our quantitative study showed that only 50% of the hypertensive patients managed at CHS in Vietnam were adherent to their medication, when we applied the 80% cut-off point for pill-days covered. There was no significant difference in adherence between patients with a high or low risk for CVD. However, adherence seemed to be influenced by age, as older patients used their medication more often in accordance with the doctor’s advice.

Adherence to hypertensive medication found in our study is similar to other studies and to results from a meta-analysis of adherence in selected databases, ranging from 26 to 78% [27, 32, 42, 43]. In our case, village health workers sometimes reminded subjects to visit the CHS for their check-up, which may have contributed to a relatively high level of adherence. A previous quantitative study in Vietnam also considered adherence of subjects during a 17 months study period. However there the adherence was measured by numbers of check-ups and the threshold applied was appearance for at least one check-up per one or two months [20], which could have led to somewhat different interpretations and results.

Increasing age was also associated with better medication adherence in our study, which is similar to previous studies on that issue [19, 27, 43–45].

The level of CVD risk has been reported as a factor influencing adherence with antihypertensive and lipid-lowering therapy [46]. We could not confirm this phenomenon in our quantitative study. The disagreement between these studies might be elucidated by differing ways to clarify CVD risk. For example, the previous study classified CVD into three levels: (i) angina or coronary angiography, (ii) coronary artery bypass graft, percutaneous transluminal coronary angioplasty or history of coronary heart disease and (iii) acute or prior myocardial infarction [46], whereas in this study we used the Asian model for risk estimation, which allows us to measure the 8-year-risk. Patients were divided into only two groups (<10% vs. ≥10% risk) [47]. In our study, physicians did not explicitly inform their patients about their level of CVD risk at baseline. Patients not knowing about their own risk level could explain why we did not find an association between level of CVD risk and adherence.

The major strength of this study is the combination of qualitative insights and quantitative analyses. Therefore, estimation of medication adherence could be based both on medical records and on data from in-depth interviews within a selected sample.

A small sample was interviewed in depth to obtain more detailed information about their adherence and the factors influencing whether they took their drugs as prescribed. The findings from the in-depth interviews indicate similar issues as were detected in previous studies [48–50], suggesting that (non-) adherence is related to awareness of risks for complications, to the presence of and wish to deal with side effects, and to absence of symptoms of high BP (“feeling healthy”). All adherent subjects mentioned that they were motivated to adhere because they were aware of potential complications. This illustrates how the qualitative study adds useful information to results from the quantitative part.

Furthermore, the qualitative results revealed that one main reason for subjects to adhere to therapy was their awareness of the seriousness of hypertension complications. These results show the importance of understanding how patients decide on and exhibit adherence to medication, and demonstrate that it is insufficient to assume that hypertension patients will just follow the prescription, as has sometimes been suggested [51].

The discrepancy between qualitative and quantitative data in classifying patients as adherent or not suggests that researchers must be careful in interpreting results from quantitative studies. Similar as in previous studies, exact measurements and analyses of drug use may result in both over- and under-estimation of adherence. Our fieldwork showed again that one has to be careful in interpreting results from quantitative study. For example, we used information on numbers of drugs prescribed, but actual use of the medication can differ largely. Potential discrepancies in adherence suggested in the quantitative and qualitative sub-studies could also be explained by the fact that in our context, patients may buy medicines for hypertension without prescription in the pharmacy. This will potentially be similar in other developing countries, which may lead to underestimation of drug use when only health-care facility data are used for estimation. Finally, we note that self-reporting may have its limitations; for example, recall bias may be an issue.

## Conclusions

Medication adherence was relatively low among hypertensive subjects in Vietnam but similar to that in many other countries. CVD risk at baseline survey did not significantly differentiate adherent from non-adherent subjects. Yet, significant differences in adherence were found for age. This may suggest that rather than risk profile, age should be considered for guiding the choice on who to target for improving medication adherence. Our qualitative study enabled further detailing of factors influencing adherence and indicated that the quantitative results should be interpreted with caution.

## Supporting information

S1 Appendix In-depth interview guideline. (RTF)

S1 Data Adherence quantitative data. (SAV)
