# Supplementary material for: Adherence to hypertension medication: Quantitative and qualitative investigations in a rural Northern Vietnamese community
Source: PLoS One. 2017 Feb 1;12(2):e0171203. doi: 10.1371/journal.pone.0171203 (PMC5287477; doi:10.1371/journal.pone.0171203)
Supplement: S1 Appendix — (RTF) [file pone.0171203.s001.rtf]

In-depth interview guideline
Adherence of hypertensive patients at CHSs

Introduction
Please be introduced myself. I am a researcher at TNUMP. As far as I know that you are hypertensive patient. So we would like to have your ideas on hypertension and using hypertensive drugs. It will take around 30 minutes for our talk. You have right to withdraw at any time during our talk.  
Administration
Name, Sex, Age, Address, marital status, living address, job? 
Hypertension and health service used
When do you start recognize that you are hypertensive patient? At what level you are in the first time? 
Why do you choose this kind of heath service? Why you do not choose the others? (Distance, convenience, good health service, good drugs….)? is it easy or difficult for you to access health care service? What make it easy/difficult?
How often do you visit health service for hypertension? Why do you go often/not often like this?
In cases, patients do not visit health services for hypertension management frequently, why don't you visit health service to manage hypertension?
Where do you get hypertension drugs? In case, patient goes to private pharmacy store to buy drugs, ask question: why don't you get drugs from health service? Who give you advice on drug use or therapy?
How much do you have to pay to buy drugs for each month? Is this expensive? Can you effort to pay? Do you consider it as a lot of money for hypertension treatment?
Do you get doctor's advice on hypertensive drugs use? Are you satisfied with advices? What kind of information do you need more in term of therapy/drug use and hypertension and its adherence? 

Hypertensive drugs used
How many pills per day do you often take? How many times per day do you often take? Count if they use frequently or forget some pills (list answers)? Show me the way to know how often you use drugs? 
Do you follow doctor's prescription or you use drugs by yourself? 
Do you forget to take drugs? Do you stop to take drugs in some days? Do you sometimes forget to take drug in the morning or afternoon? How often is it (ask this with all about questions)? 
Do you change drug therapy, such as change number of pills per day or change name of drugs? You changed by yourself or doctor changed the therapy? Why do you have to change drugs or therapy?
In what way helps you to remember take drugs every day? Is there anyone who helps you to remember taking drugs frequently?
Is the therapy control your blood pressure level? What kinds of drug do you like/don't like and why? 
Do you get any side effect (problem) from taking drugs? If yes, what are they? How do you react when side effect occur, such as stop using drugs or go to see doctor or change drug by yourself…?
Do you know any herbal medicine to treat hypertension? Do you use them? Why do you use them? Is this helpful? Do you prefer herbal medicine or Western medicine? 
Do you meet any difficulties in taking drugs? In what way, it will help you using drug easily/difficulty? What skills do you need to take drugs often as doctor's prescription? 
Do you know any problem it may causes to you if you do not take drugs or do not take drugs frequently? What are they?
When you stop or forget to take drug, is there any problem which may cause to you?
Sometimes you forgot to take drug or did not take drug actively, why is it? What factors lead you to adherence or non-adherence with drug therapy? 
What factors can help you to improve the adherence with the therapy?
What do you expect from health service to help you improve the adherence with the therapy?
Do you have any questions that may help you now?
Thank you very much for your time to answer our question!
